# New Insights on the Early Interaction Between Typhoid and Non-typhoid *Salmonella* Serovars and the Host Cells

**DOI:** 10.3389/fmicb.2021.647044

**Published:** 2021-07-01

**Authors:** Bárbara M. Schultz, Felipe Melo-Gonzalez, Geraldyne A. Salazar, Bárbara N. Porto, Claudia A. Riedel, Alexis M. Kalergis, Susan M. Bueno

**Affiliations:** ^1^Departamento de Genética Molecular y Microbiología, Facultad de Ciencias Biológicas, Pontificia Universidad Católica de Chile, Santiago, Chile; ^2^Laboratory of Clinical and Experimental Immunology, School of Medicine, Pontifical Catholic University of Rio Grande do Sul, Porto Alegre, Brazil; ^3^Program in Translational Medicine, Hospital for Sick Children, Toronto, ON, Canada; ^4^Departamento de Ciencias Biológicas, Facultad de Ciencias de la Vida, Millennium Institute on Immunology and Immunotherapy, Universidad Andrés Bello, Santiago, Chile; ^5^Departamento de Endocrinología, Facultad de Medicina, Pontificia Universidad Católica de Chile, Santiago, Chile

**Keywords:** *Salmonella enterica*, *Salmonella enterica* ser. Typhimurium, *Salmonella* pathogenesis Island (SPI), virulence factors, inflammasome, autophagy, organoid

## Abstract

*Salmonella enterica* is a common source of food and water-borne infections, causing a wide range of clinical ailments in both human and animal hosts. Immunity to *Salmonella* involves an interplay between different immune responses, which are rapidly initiated to control bacterial burden. However, *Salmonella* has developed several strategies to evade and modulate the host immune responses. In this sense, the main knowledge about the pathogenicity of this bacterium has been obtained by the study of mouse models with non-typhoidal serovars. However, this knowledge is not representative of all the pathologies caused by non-typhoidal serovars in the human. Here we review the most important features of typhoidal and non-typhoidal serovars and the diseases they cause in the human host, describing the virulence mechanisms used by these pathogens that have been identified in different models of infection.

## Introduction

*Salmonella enterica* (*S. enterica*) is a Gram-negative, non-spore-forming, facultative, anaerobic bacterium that belongs to the *Enterobacteriaceae* family (Fàbrega and Vila, 2013). Over 2,500 serovars of *S. enterica* have been identified and most serovars infect a broad range of vertebrate animals. Only a few of them are host-specific, being divided into human-restricted typhoidal serovars such as the causative agent of typhoid fever (*S. enterica* serovar Typhi and Paratyphi), and non-typhoidal *Salmonella* (Wain et al., 2015). The non-typhoidal serovars (NTS), have been linked to infection of a variety of hosts, frequently zoonotic, causing acute and self-limiting gastroenteritis that can commonly cause foodborne illness in humans (Scallan et al., 2011). NTS constitutes a common health problem accounting for about 93.7 million cases per year and 155,000 deaths (Majowicz et al., 2010; Ao et al., 2015). Furthermore, these ailments are the second cause of diarrheal death in children younger than 5 years old, which has global public health relevance (Scallan et al., 2011; Lanata et al., 2013). Although infections by NTS are generally self-limited, immunocompromised patients can present extra-intestinal complications and develop chronic carrier states, which have been implicated in invasive non-typhoid *Salmonella* (iNTS) (Ao et al., 2015; Stanaway et al., 2019a). *Salmonella enteric*a serovar Typhimurium (*S.* Typhimurium) and *Salmonella enterica* serovar Enteritidis (*S.* Enteritidis) are the two serovars commonly isolated of NTS and iNTS (Dekker et al., 2018; Mather et al., 2018; Williamson et al., 2018).

In this review we will focus on the pathogenesis of typhoidal and non-typhoidal serovars, the virulence factors used by them and the immune response generated, mainly in studies performed in human cell lines, comparing these responses with what occurs in typhoid fever caused by *S*. Typhi. In addition, as the extensive knowledge about one of the NTS, *S*. Typhimurium, is due to the studies performed in mouse models, we will discuss whether these effects have also been proven in studies in human models and also if these mechanisms are conserved in typhoidal serovars.

## Invasive Diseases Caused by *Salmonella* Serovars

### Typhoidal *Salmonella* Serovars

Few serovars of *S. enterica* are adapted to the human, which is the only reservoir for this infection. Enteric fever caused by *S*. Typhi and *S*. Paratyphi A, B, and C, is a systemic infection that can be severe and even life-threatening (Wain et al., 2015). The incubation period is 6–30 days, with gradually increasing fatigue and fever. If the symptoms are not treated, serious complications appear between 2 and 3 weeks of illness, as intestinal hemorrhage or perforation. In addition, between 10 and 15% of patients can develop severe disease, which has been associated in some studies with the age of the patient (Parry et al., 2014) and with the days of hospitalization (Cruz Espinoza et al., 2019). In 2017, 14.3 million cases were identified worldwide, with 135,000 deaths caused by enteric fever, which shows a decrease of 44.6 and 41%, respectively, as compared to 1990. The decrease in the number of cases was mainly due to the improvement in the sanitization, water supply, food handling, and an increased access to antibiotics treatment (Stanaway et al., 2019b). However, in children and lower-income countries from Asia, sub-Saharan Africa, and Oceania, the overall case-mortality rate with early and appropriate treatment is 0.95% (Stanaway et al., 2019b).

*Salmonella* Typhi is the leading causative agent of enteric fever. However, a switch of serovars has been observed in some places where typhoid fever is endemic, possibly due to the widespread use of the *S*. Typhi vaccine (Sudeepa Kumar et al., 2013). This has important implications for public health as the oral Ty21a vaccine strain and the parenteral Vi typhoid vaccine offers limited or no protection against *S.* Paratyphi A (Pakkanen et al., 2014; Wahid et al., 2015). Although both serovars can cause an indistinguishable disease with fever, nausea, and abdominal problems, the infection caused by *S*. Typhi generates more frequent abdominal pain and diarrhea (Maskey et al., 2006; Karkey et al., 2013). Furthermore, *S.* Typhi is associated with severe symptoms as compared to *S.* Paratyphi (Browne et al., 2020; Kheng et al., 2020), and also display extensive drug resistance to the majority of drugs available to treat typhoid fever (Klemm et al., 2018). It has also been described that both serovars differ on the route of transmission: *S.* Typhi is more related to poor water supply, and poor socioeconomic status, whereas *S.* Paratyphi is related to food consumption habits and migratory status (Karkey et al., 2013). The disease caused by both serovars is clinically indistinguishable, and its host adaptation may be the result of convergent evolution with recombination of at least a quarter of their genome (Didelot et al., 2007). Additionally, the appearance of several pseudogenes can be responsible for the differences observed between both serovars (Holt et al., 2009). Pseudogenes are genes that have lost their function by point mutation, insertion, deletions, misplaced stop codon, among others (Tutar, 2012). However, they have been retained in the genome because they regulate different process of the parent gene and, in some cases, allow the generation of new genes (Tutar, 2012).

Although typhoid fever is an important disease, especially in low-income countries, the availability of two vaccines and treatments and improved strategies to prevent the disease have lessen its spread. This is an important difference with NTS, which have little epidemiological follow-up and lack of vaccines to prevent these diseases, even when they can generate higher mortality than typhoid serovars.

### Invasive Non-typhoidal Salmonellosis (iNTS)

Invasive non-typhoidal salmonellosis (iNTS) is an invasive disease caused mainly by *S*. Typhimurium and *S*. Enteritidis infection (Gordon et al., 2008, 2010; Ao et al., 2015; Uche et al., 2017), that usually shows no gastroenteritis symptoms but fever, hepatosplenomegaly, respiratory symptoms, and septicemia (Stuart and Pullen, 1946; Olsen et al., 2003). More than half of iNTS cases globally are produced in sub-Saharan Africa, and according to Ao et al., iNTS can cause 3.4 million cases and more than 600,000 deaths per year. Although there are some differences in the numbers as compared to those analyzed by Ao et al. (2015) and Stanaway et al. (2019a), both studies agreed that iNTS are more deadly than NTS, having 25 and 1–5% case risk mortality, respectively (Ao et al., 2015). However, depending on the patient, iNTS can reach up to 72% (Uche et al., 2017), despite antibiotic treatment (Mandomando et al., 2015). Even so, the mortality rate of invasive infection caused by NTS is higher than the infection caused by *S*. Typhi, being the occurrence of MDR also higher than the typhoidal serovar (Shahunja et al., 2015). In recent years in Africa, the invasive Sequence Type 313 (ST313) of *S*. Typhimurium has been more prevalent than ST19 (Mandomando et al., 2015), the commonly zoonotic worldwide agent of gastroenteritis. ST313 has also expanded worldwide and is now found in the United Kingdom (Ashton et al., 2017) and Brazil (Seribelli et al., 2020). Although the genome of ST313 and ST19 are 95% identical (Canals et al., 2019), ST313 had undergone genome degradation, as is observed in the host-adapted *S*. Typhi. ST313 that has suffered gene acquisition (new phage elements encoding antibiotic resistance genes), deletion and addition of pseudogenes, and appearance of single nucleotide polymorphisms (SNPs) affecting metabolic genes (Kingsley et al., 2009; Okoro et al., 2015), which suggest a further specialization to a narrow human niche as immunocompromised humans (Van Puyvelde et al., 2019). All of these genetic changes make ST313 evolutionarily distant from ST19 (Okoro et al., 2015). According to the genomic change, it seems that ST313 is not transmitted zoonotically or environmentally, but rather due to contact with another infected human. Therefore, the iNTS carriage, as described below, could be an important reservoir for human-human transmission (Kariuki et al., 2006).

All the genetic modifications detected in iNTS serovars could involve in a distinct interaction with the host: ST313 induces an intermediate response, is more invasive and inflammatory than *S*. Typhi, but less than *S.* Typhimurium ST19 (Carden et al., 2015; Ramachandran et al., 2015). Studies in human cell lines have attributed this phenotype to a lower expression of the *Salmonella* effector protein SopE2 and the flagellin fliC, which could result in less intestinal invasion and less inflammatory responses due to inflammasome activation, caspase-1-dependent macrophage death, IL-1β release, and neutrophil infiltration (Carden et al., 2015). Accordingly, ST313 can survive and replicate inside both human and murine macrophages at a higher rate, being more resistant to killing than ST19 (Ramachandran et al., 2015). Thus, ST313 has developed efficient strategies to evade the host immune response, which are distinct to those displayed by ST19 and *S.* Typhi.

### Carriage State in Typhoid and iNTS

Between 2 and 5% of the typhoid fever patients cannot eliminate the bacteria and they serve as a reservoir of the bacteria and spread the infection (Levine et al., 1982). Even more, 25% of carriers are asymptomatic (Levine et al., 1982). In addition to the existence of the vaccines against *S*. Typhi, the asymptomatic carriage state in the gallbladder makes it challenging to eradicate the disease in endemic areas due to intermittent shedding and in consequence human-human transmission. Although there is not a gold standard methodology to diagnose chronic carriage, some diagnostic methods have been indeed developed to detect asymptomatic carriers. For instance, Näsström et al. (2018) identified five metabolites in the plasma of typhoid fever patients, allowing the distinction between the carriage and non-carriage patient, but not between the carriage state of *S*. Typhi and Paratyphi. This would be an excellent approximation to decrease the dissemination of the pathogen (Näsström et al., 2018). Tracing by a diagnostic method is even more critical because the infection and carriage state of *S*. Typhi have been correlated with carcinoma of the gallbladder (Nath et al., 1997).

In contrast, the carriage stage caused by *S.* Typhimurium infection is usually observed in immunocompromised patients, although this can also happen in immunocompetent patients. Carriers can excrete bacteria and are an essential factor in the transmission of *S*. Typhimurium, because the median duration of this excretion can be 4–5 weeks, with the most extended duration being 279 days after the infection (Octavia et al., 2015). In this sense, a study including patients from Israel shows that 2.2% of the infected patients presents long term infection that lasts for more than 30 days, whom can become human reservoirs for NTS transmission (Marzel et al., 2016). Of this 2.2% infected patient, a 93% were immunocompetent and 65% were symptomatic with relapsing diarrhea, suggesting a persistent manifestation, distinct from the known asymptomatic carriage of typhoidal *Salmonella* Moreover, *S.* Typhimurium can also cause an asymptomatic carriage state of NTS infection. This was assessed in three different studies which reported percentages of 7.7% (Sirinavin et al., 2004), 6.9% (Kariuki et al., 2006), and 2.4% (Im et al., 2016) of carriage without symptoms. In this sense, a study performed by Bakkeren et al. (2019) observed in mouse models that mucosa-associated persisters of *S.* Typhimurium can serve as a reservoir to relapsing diseases and spread a plasmid carrying antibiotic resistance that is of clinical importance. This implies that humans can also have an essential role in the epidemiology of community-acquired NTS and probably iNTS.

### Genome Differences Between *S*. Typhi and *S.* Typhimurium

*Salmonella* Typhi and *S*. Typhimurium share among 80% of their genome (Tang et al., 2013). Nevertheless, these two serovars can cause different disease, host–pathogen interactions and immune responses, which can be attribute to approximately 600 and 480 unique genes, respectively (Parkhill et al., 2001). Indeed, only 6% of those common genes have 100% sequence identity (Tang et al., 2013). Additionally, *S*. Typhi has suffered loss of different gene functions and presents several pseudogenes (McClelland et al., 2001), consistent with the niche specialization, as in the case of the invasive *Salmonella* ST313.

A proteomic study has shown a very similar proteomic profile between these two serovars, revealing that from a total of 1,506 shared proteins, just seven of them were encoded by genes present on SPIs, and just a few proteins were expressed in a unique serovar (85 proteins were expressed in *S*. Typhimurium and 36 in *S*. Typhi) (Wang et al., 2014). Also, both serovars do not exhibit differentially expressed SPI-encoded proteins. In contrast, flagella and chemotaxis proteins are down regulated in *S*. Typhi, which can be due to its colonization niche: systemic in the case of *S*. Typhi and restricted to the gut in *S*. Typhimurium. Finally, this study shows that both strains have different metabolic capabilities in carbohydrates and amino acid usage, which can be implicated in the different pathology caused in humans (Wang et al., 2014). In this sense, there are around 88 specific typhoidal family proteins related to Vi polysaccharide capsule biosynthesis, export protein, and pilus assembly that are not present in *S*. Typhimurium (Zou et al., 2014). Consistent with this, the Vi polysaccharide capsule is essential to suppress the inflammatory process induced in the intestinal epithelium, decreasing IL-8 secretion and the influx of neutrophils, which allows the systemic dissemination (Raffatellu et al., 2005a).

The adhesiome of *S*. Typhimurium includes at least 12 chaperone-usher fimbriae, among other important adhesins and non-fimbrial adhesins, which are essential for long-term colonization in mice. However, they are not well characterized as these genes are not well expressed in laboratory conditions (Hansmeier et al., 2017). In the case of *S.* Typhi, 14 fimbrial gene clusters, including 12 chaperone/usher fimbria, have been identified. Type I fimbria is one of these chaperone/usher fimbriae and, as in *S*. Typhimurium, is also involved in adhesion and invasion of human epithelial cells and is related to phagocytosis by THP-1 cells. It is important to mention that the bacterial adhesion protein FimH has point mutations in *S.* Typhi that allow the specificity of this serovar for human cells (Dufresne et al., 2018). According to the above, another chaperone/usher fimbrial operon denominated stg, absent in other serovars, is expressed during infection of human macrophages and is also related to the specific adhesion to human cells (Forest et al., 2007). On the other hand, depending on the strain, *S.* Typhimurium present different plasmids, one of them is the virulence plasmid designated as pSLT, which under optimal condition is self-transmissible and it is related to the systemic phase of disease. However, this plasmid evolved and is maintained in the host cell by vertical rather than horizontal transmission and can suffer different changes under the influence of transposons, insertions and hybridation with other plasmids (Hiley et al., 2019). It was thought that genes within pSLT were absent in *S.* Typhi and that this serovar presented just the pR(ST98), a conjugative plasmid with MDR. However, it was described that an avirulent *S*. Typhimurium carrying pR(ST98) induced higher caspase-mediated apoptosis and bacterial survival in murine macrophages (Wu et al., 2010). This effect can be mediated by *spvR*, a transcriptional regulator of its own gene and the *spv*ABCD operon (Krause et al., 1995), and *spvB*, which prevents the actin polymerization (Lesnick et al., 2001). These two genes are encoded by this chimeric plasmid, which exhibit 99% homology with the genes present in pSLT (Huang et al., 2005). In the case of ST313, Kingsley et al. identified at least four different plasmids, one of them, pSLT-BT, closely related to the pSLT. pSLT-Bt present a composite Tn21-like mobile element, in which are encoded all the determinants that confer multiple antibiotic resistance associated with the virulence and the invasion of this serotype, and this element would act as a platform to the acquisition of new virulence genes (Kingsley et al., 2009).

Several genes encoding important effector proteins present in *S.* Typhimurium are absent or are pseudogenes in *S.* Typhi. In the case of *S*. Typhi, the pseudogenization of functional genes is an active process, which has been related as the main driving force in evolution in the case of this serovar, as mentioned before (Baddam et al., 2014). It is believed that loss of gene function or gene deletion are related to the niche specialization and probably accounts for the differences observed in the pathology in *S*. Typhi. However, the exactly role of the pseudogene remains elusive (Baddam et al., 2014). In this sense, a study performed by Eswarappa et al. (2008) showed that SipD and SptP (effector proteins of SPI-1) and SseC, SseD, SseF, and SifA (effector proteins of SPI-2) evolved differentially between both serovars. It also described that the effector proteins sspH and steB of SPI-1 and SseL, SseK1/2 and3 of SPI-2 are missing in *S*. Typhi, whereas others as SopA, SopE2, SopD2 and SseJ are pseudogenes [reviewed in Sabbagh et al. (2010)]. One of the most important effector proteins is SptP, which is expressed by *S.* Typhimurium and is absent in *S*. Typhi. During infection with *S.* Typhimurium, SptP helps in reversing the cytoskeleton changes in order to prevent the activation of the immune response by an excessive activation of the effector SopE (Keestra et al., 2013) and its production affect the immune response mediated by mast cells (Choi et al., 2013). *S.* Typhimurium lacking SptP are less virulent and less invasive in HeLa cells. However, SptP is absent in *S*. Typhi and SopE is non-essential for the cellular invasion of this serovar (Johnson et al., 2017), suggesting that virulence of *S.* Typhi relies in different mechanism to cause cell invasion. Along the same lines, Hannemann and Galá (2017) described that a differential reprogramming in the transcriptional profile of intestinal epithelial cells occurs during the infection with typhoidal and non-typhoidal serovars, which is dependent of SPI-1 encoded TTSS and serovar-specific effector proteins secreted to the host cell. This study shows that both serovars of *Salmonella* activate different transcriptional profiles, which are related to the specific effector proteins secreted during invasion that results in a differential inflammatory response generated by the infection (Hannemann and Galá, 2017).

One important virulence factor only present in *S*. Typhi and absent in *S.* Typhimurium is the polysaccharide capsule known as Vi antigen, which interfere with the innate immune response avoiding, for example, the phagocytosis and oxidative burst (Kossack et al., 1981). This capsule is regulated by two separated loci, *viaA* and *viaB;* the stable expression of this capsule depends on *viaB* locus (Kolyva et al., 1992). A positive regulator of the *viaB* locus, TviA, is only present in *S.* Typhi and is absent in other serovars of *Salmonella* and mediates the evasion of inflammasome activation and pyroptosis in the human monocytic cell line THP-1 by decreasing flagellin expression (Winter et al., 2015). In addition, heterologous expression of TviA in *S.* Typhimurium also reduces inflammasome activation, indicating that this mechanism may be involved in *S*. Typhi survival in humans and typhoidal disease (TD) pathogenesis (Winter et al., 2015). Another important virulence factor of *S*. Typhi is the typhoid toxin, which is an AB toxin being the subunit CdtB responsible of the toxic activity. This toxin is specific of human cells as it binds to sialoglycans terminated in acetyl neuraminic acid but not those glycans present in other mammals (Song et al., 2013; Deng et al., 2014). This toxin is expressed when the bacterium is inside the SCV, is transported to the nucleus by vesicles inducing DNA damage and cell cycle arrest, which in turn induces cytoplasmic distention and nuclear enlargement (Lara-Tejero and Galán, 2002; Haghjoo and Galán, 2004). More differences between both strains are reviewed in Johnson et al. (2017).

Some functions related to virulence genes encoded on SPI are also responsible for the differences observed in clinical manifestations between both serovars. As mentioned above, *S.* Typhimurium can interfere with vacuole and lysosome colocalization and avoid the proper antigen presentation by dendritic cells (DC). In this sense, a comparative study between *S*. Typhi, *S.* Typhimurium, and *S*. Enteritidis shows that both *S.* Typhi and Enteritidis failed to avoid the antigen presentation in mouse DC, are unable to survive inside them, and in consequence activate the T cell response (Bueno et al., 2008). In contrast, only *S*. Typhi can replicate and survive inside human DC (Bueno et al., 2008). In this line, it has been shown that peritoneal murine macrophages clear more effectively *S*. Typhi than *S*. Typhimurium in *in vivo* experiments, which was not reproduced *in vitro*, implying that the serovar-specific macrophage killing depends on the interactions present *in vivo* or it is affected by paracrine soluble factors (Xu et al., 2009). Finally, Spanò and Galán (2012) shows that the expression of T3SS effector protein GtgE of *S.* Typhimurium in *S.* Typhi allows its survival and replication inside murine macrophages. All these studies have helped us to understand in a proper manner how the infective cycle of S. Typhi occurs; however, a suitable model is needed to study the host specificity of this serovar.

## Non-Typhoidal *Salmonella* Serovars

*Salmonella* Typhimurium is one of the most prevalent serovars of NTS worldwide (Reddy et al., 2010; Dekker et al., 2018; Mather et al., 2018; Williamson et al., 2018), causing salmonellosis through the consumption of contaminated food or water. The clinical manifestations include abdominal pain, diarrhea, nausea, vomiting, fever, and headaches, lasting less than 10 days, and are usually resolved with rehydration treatment with fluids and electrolytes. However, iNTS in immunocompromised patients, multi-drug resistance (MDR), and carrier stage (Marzel et al., 2016), are common complications of *S*. Typhimurium infection.

### Virulence Factors of *S.* Typhimurium Involved in NTS

The clinical symptoms observed in NTS are generated due to several virulence factors present in the genome of *S.* Typhimurium, encoded in at least 23 *Salmonella* Pathogenicity Islands (SPIs) (Blondel et al., 2009; Desai et al., 2013; Hayward et al., 2013). Various serovars share only five of these SPIs, being the SPI-1 and SPI-2 the most important to the infective cycle. Both SPIs encode Type Three Secretion Systems (T3SS) that allow contact-dependent translocation of a set of effector proteins into the eukaryotic cytoplasm (Hansen-Wester and Hensel, 2001; Moest and Méresse, 2013; Pezoa et al., 2013). Both SPI present several effector proteins, and those that can be important to the course of the infection and have been evaluated in human cell lines will be described below (Table 1).

**TABLE 1:** Virulence factor of S. Typhimurium, their location and main function in human and murine models.

Virulence factor	Location	Function	References
*yqiC gene*	Non-SPI	• Suppresses type-1 fimbriae over-expression, regulates flagella and motility in human IECs. • Regulates gene expression of SPI-1 and SPI-2.	Wang et al., 2016
*SipA*	SPI-1	• Allows the invasion in mouse and human IECs. • Allows the hyper-replicative state in mouse and HeLa cells. • Induces the transepithelial migration of neutrophils.	Zhou et al., 1999; Silva et al., 2004; Knodler et al., 2014b; Chong et al., 2019
*SopE2*	SPI-1	• Allows the penetration and invasion of IECs in mouse model. • Allows the expression of SPI-2 in murine IECs. • Exacerbates the intestinal inflammation on human IECs.	Bruno et al., 2009; Zhang et al., 2018
*SopB*	SPI-1	• Important for intracellular replication and expression of SPI-2. • Avoids the apoptosis of human epithelial cells. • Involved in the SCV formation on human IECs. • Exacerbates the intestinal inflammation on human IECs.	Bruno et al., 2009; Ruan et al., 2016; Zhang et al., 2018; Stévenin et al., 2019
*SopF*	SPI-1	• Blocks *S*. Typhimurium-induced autophagy in He-La cells	Xu et al., 2019
*SpiC*	SPI-1	Participates in the regulation of the exocytic pathway in human IECs.	Hallstrom and McCormick, 2016
*SptP*	SPI-1	Avoid immune response mediated by neutrophils in human and mouse mast cells.	Choi et al., 2013
*SpeG*	SPI-1	• Regulates the expression of motility-related genes in HeLa cells. • Involved in intracellular replication in human non-phagocytic cells.	Fang et al., 2017
*SifA*	SPI-2	• SIF formation. • Modulates antigen presentation in mice. • Affects the accumulation of HLA-DM in the intracellular in mice.	Mitchell et al., 2004; Halici et al., 2008; Knuff-Janzen et al., 2020
*SopD2*	SPI-2	• SIF formation and maturation of SCV. • Modulates antigen presentation in mice.	Halici et al., 2008; Knuff-Janzen et al., 2020)
*SseJ*	SPI-2	SIF formation and maturation of SCV.	Knuff-Janzen et al., 2020
*SseF*	SPI-2	Contributes to the intracellular replication and block the autophagosome formation in human IECs.	Yu et al., 2016; Feng et al., 2018
*SseG*	SPI-2	Contributes to the intracellular replication and block the autophagosome formation in human IECs.	Yu et al., 2016; Feng et al., 2018
*SseK*	SPI-2	• Avoids the apoptosis of the cells in HeLa cells. • Avoids the necroptotic dead in murine cells.	Günster et al., 2017
*Ssel*	SPI-2	• Inhibits the migration of DC in mouse models. • Inhibits the autophagy allowing the intracellular replication in HeLa cells.	McLaughlin et al., 2009; Mesquita et al., 2012
*SteD*	SPI-2	• Favors the ubiquitination of MHC-II in murine models. • Favors the degradation of MHC-II in Mel JuSo cells.	Bayer-Santos et al., 2016

#### SPI-1 Related Effector Proteins

SPI-1 facilitates the entry to non-phagocytes cells, as enterocytes (Raffatellu et al., 2005b). However, this is not the only function that this SPI presents [reviewed in Lou et al. (2019)]: (1) it is involved in attachment, invasion, and replication inside cells in murine models; (2) avoids apoptosis of intestinal epithelial cells (IECs) (Ruan et al., 2016; Zhang et al., 2018), and (3) suppresses the host immune response during intestinal inflammation (Hapfelmeier et al., 2005; Choi et al., 2013). SPI-1 mediates the binding to the host cell, allowing the secretion of effector proteins by the T3SS encoded by SPI-1 (T3SS-1) that induce, among other effects, the actin polymerization that leads to membrane ruffles observed in human and murine models (Zhou et al., 1999; Hallstrom and McCormick, 2016). Furthermore, the type I fimbriae also have an essential role in the attachment and invasion, mediating a reversible binding with the IECs, as shown in human intestinal cell lines. In this sense, *S.* Typhimurium employs an important non-SPI gene, *yqiC*, to suppress type-1 fimbriae over-expression, regulating flagella and motility, and also is involved in the regulation of SPI-1 and SPI-2 genes expression in human IECs (Wang et al., 2016).

Several studies have postulated *Salmonella* invasion protein A (SipA) as an important effector protein in both human and murine models of infection. In a neonate mouse model, it was reported that SPI-1 effector proteins such as SopE2 and SipA have a redundant role, but are necessary for the penetration and invasion of the epithelial barrier (Zhang et al., 2018). Also, the expression of SipA with SopE2/SopB is necessary for the generation of the replicative intraepithelial endosomal compartment, crucial for replication and expression of SPI-2 virulence genes in murine IECs (Zhang et al., 2018). Additionally, it has been described that *S*. Typhimurium can escape from the *Salmonella* containing vacuole (SCV), which is mediated mainly by genes encoded in SPI-1 (Knodler et al., 2014b), and replicate in the cytosol, a state called hyper-replicative (Knodler et al., 2014b). In this state, SipA is required for cytosolic replication and avoids the SCV degradation in HeLa cells by autophagy (a host mechanism present in IECs and multiple immune cells which will be discussed in detail in Section Crosstalk Between IECs and Innate Immune Cells During *Salmonella* Infection) (Chong et al., 2019). Knodler et al. (2014b) found the presence of cytosolic bacteria in different human epithelial cell lines and showed that at least half of the infected cells presented cytosolic bacteria at 7 h post-infection, which is in agreement with the SipA function. However, it is possible that autophagy can have a time-dependent function. Moreover, SipA can also favor *Salmonella* invasion in human IECs. *S.* Typhimurium modulates the distribution of tight junction proteins in a monolayer of T84 intestinal cells, with the concomitant possibility of disseminating and allowing the transmigration of neutrophils to the site of infection (Köhler et al., 2007). In this line, it was observed that SipA allows activation of the protein kinase C (PKC)-a, which is involved in the signal transduction pathway to induce the transepithelial migration of neutrophils (Silva et al., 2004). Furthermore, SipA together with SipC participate in the regulation of the exocytic pathway in human IECs and subsequently induces the accumulation of a host membrane protein, PERP, at the apical side of epithelial cells, allowing the formation of the membrane ruffles and the infection of the cell (Hallstrom and McCormick, 2016).

Another SPI-1 essential effector protein is SopB, which avoids the apoptosis of human epithelial cells due to the inhibition of the mitochondrial ROS production generated by the typical response to the infection (Ruan et al., 2016). SopB is also a regulatory protein of the SCV formation process, as can promote the fusion of SCV-IAM (infection-associated macropinosomes), and also can trigger its rupture due to the formation of membrane tubules called spacious vacuole-associated tubules (SVAT) in human IECs (Stévenin et al., 2019). Also, SopB exhibit a redundant role with the SPI-1 effector proteins SopE and SopE2, exacerbating intestinal inflammation. This is due to the production of inflammatory mediators and the stimulation of the Rho-family GTPases in human epithelial cells, which subsequently activates the mitogen-activated protein (MAP) kinase and NF-κβ signaling (Bruno et al., 2009). SptP is another important SPI-1 effector protein for NTS, which is a tyrosine phosphatase secreted into the cytosol of host cells. It has been described that in both mouse and human mast cells SptP dephosphorylates two proteins required for the degranulation and, in consequence, decrease the recruitment of neutrophils to the site of infection, preventing NTS bacterial clearance (Choi et al., 2013).

SpeG is another SPI-1 gene involved in the intracellular replication of NTS and has been tested in different human non-phagocytic cell lines with the same results (Fang et al., 2017). In HeLa cells, speG downregulates the expression of genes related to flagellar biosynthesis and fimbria expression, which affects considerably the motility of the bacteria (Fang et al., 2017). It has been seen that the addition of polyamides to the culture medium decreases the motility of the *S.* Typhimurium WT strain (Fang et al., 2017), indicating that speG exerts the regulation of polyamine metabolism genes to decrease its antimicrobial function (Fang et al., 2017). Therefore, speG can modulate both host and *S.* Typhimurium genes in order to favor bacterial survival.

#### SPI-2 Related Effector Proteins

The bacteria inside SCV express SPI-2 genes, which as SPI-1, present a variety of functions such as maintaining the SCV membrane, regulating intracellular SCV positioning, forming the membranous filament-like extensions that radiate outward from the SCV -termed *Salmonella*-induced filaments (SIFs)- and replication inside murine and THP-1 cells (Knuff-Janzen et al., 2020). All these functions are mediated by the TTSS-2 ability to translocate effector proteins across the SCV. It was believed that the formation of SIF was mediated exclusively by SifA; nevertheless, strains lacking this protein can also form this structure. It is probable that the interaction between SifA, SopD2, and SseJ, which are critical for the maturation of SCV, mediate the formation of SIF (Knuff-Janzen et al., 2020). Other important proteins of SPI-2 are SseF and SseG, which are located in the SCV membrane (Yu et al., 2016) that mediate the SCV-Golgi association with other host proteins such as the acyl-CoA binding domain containing 3 (ACBD3). These interactions, mainly with Rab1A, allow the intracellular replication inside human IECs by blocking the early steps of autophagosome formation (Yu et al., 2016; Feng et al., 2018).

SPI-2 effector proteins are also needed by *Salmonella* to survive and replicate inside the phagocytic cells. *S.* Typhimurium replication inside THP-1 monocytes depends on the action of multiple effectors proteins and further studies are necessary to elucidate the function of each specific protein (Knuff-Janzen et al., 2020). So far, it is known that in HeLa cells, SseK protein has an additive effect on the inhibition of TNF-α-dependent NF-κβ activation (Günster et al., 2017), avoiding the apoptosis of the cell. However, in murine RAW264.7 macrophages, this protein avoids the necroptotic death, suggesting that SseK effector protein has different host cell targets (Günster et al., 2017). It is important to mention that the apoptosis analyses have not been performed in human macrophages and thus further studies are required to confirm SseK regulation of apoptosis in humans.

The migration of DC and macrophages is important to generate a systemic disease. However, in mouse models, it has been seen that the SPI-2 effector protein Ssel inhibits this process, allowing the long-term infection and avoiding the proper clearance of systemic bacteria in a mouse model (McLaughlin et al., 2009). Interestingly, the invasive ST313 lost Ssel gene due to the genomic degradation, which allows the infection of migratory DC CD11b^+^ and the systemic dissemination (Carden et al., 2017). Nevertheless, another study shows that Ssel is not the only effector protein related to the inhibition of DC migration, but is unknown whether these proteins perform this process together or independently (Mclaughlin et al., 2014). In human cell lines, this particular function has not been evaluated. However, it was reported in HeLa cells that Ssel inhibits the recruitment of autophagy markers and, consequently, allows the replication inside the vacuole, effect that was also observed in RAW264.7 cells (Mesquita et al., 2012).

SPI-2 is also able to modulate antigen presentation on host cells, which was evaluated in mouse bone marrow-derived DC, where SifA, SspH2, SlrP, PipB2, and SopD2 were equally important at interfering with the fusion of processed peptides and the compartments that contain MHC-II complexes (Halici et al., 2008). In this line, SteD mediates the ubiquitination of MHC-II by allowing its fusion with vesicles containing the E3 ubiquitin ligase MARCH8 and, consequently, the degradation of the MHC-II (Bayer-Santos et al., 2016). Furthermore, SPI-2 and SpiC protein are critical for the evasion of antigen presentation by mediating the survival of *S*. Typhimurium in vacuoles that lack lysosomal markers that are not degraded, thus the bacterial proteins are not present in MHC context (Tobar et al., 2006). In the case of human cell lines, it was observed that in infected Mel JuSo cells, Human Leukocyte Antigen-DM (HLA-DM), an intracellular protein involved in the antigen presentation, was synthesized, assembled, transported to peptide-loading compartments, and loaded with peptide, in normal levels. However, HLA-DM accumulates in the intracellular compartment, possibly due to interference with normal trafficking caused by *Salmonella* infection. In line with the results in mouse models, this effect was also mediated by SifA (Mitchell et al., 2004). Thus, it is possible to conclude that SPI-2 effector proteins exhibit conserved functions across human and mouse IEC and phagocytic cells during infection.

### Differences Between Mouse and Human Models to Study *S*. Typhimurium Infection

Mouse models are an indispensable tool to study the pathology of human disease and allow the identification of several factors that can be targeted for treatment or vaccines in humans. However, not all human infections are replicable in animals: in the case of *S*. Typhimurium infection, although it can infect a broad range of animals, it does not generate the same disease in all of them. As the main aim of this review is to explore new findings in human models of infections and compare them to the literature existing in mouse models, we will briefly describe some of the most commonly used mouse models of infection, including immunocompetent mice, antibiotic-treated mice and immunodeficient mice.

The models of *Salmonella* infection can be divided into the study of invasive disease or gastroenteritis. *S*. Typhimurium can infect mice causing systemic disease that mimics the acute phase of typhoid fever and can target the gut-associated lymphatic tissues to generate bacteremia. In this case, two mouse models have been used: C57BL/6 wild type (WT) strain, which presents a mutation in a gene involved in the intracellular killing of the bacteria known as NRAMP-1 encoded in human by the *SLC11A1* gene allowing the development of systemic infection without intestinal inflammation (Vidal et al., 1995); and 129 × 1/svj strain that expresses a fully functional NRAMP-1 gene, which is used to study the long-term persistence observed in humans infected with *S*. Typhi (Monack et al., 2004). However, as mentioned above, *S.* Typhimurium can also persist in C57BL/6 mice infected with *S*. Typhimurium after antibiotic treatment (Schultz et al., 2018) as well as in humans (Marzel et al., 2016).

In humans, *S.* Typhimurium causes gastroenteritis in the majority of the infections, accompanied with intestinal inflammation, but in mice there is no sign of intestinal inflammation because of the natural resistance provided by the microbiota (Jacobson et al., 2018). In this context, the streptomycin pre-treated mouse model has been established to evaluate the colitis induced by *S.* Typhimurium infection (Barthel et al., 2003). Concomitant with the elimination of the microbiota, genes encoded in SPI-1 cause epithelial ulceration, edema, massive infiltration of polymorphous nuclear cells, among other features that resemble the enteric infection (Barthel et al., 2003). However, in this murine model, the intestinal inflammation is generated in cecum and colon, while in humans it is mainly produced in the small and large intestine. It is crucial to keep in mind that the immune system between humans and mice are quite similar. However, differences in some processes or specific immune cell activation exist between them, determining the different immune responses.

Although the infection with *S.* Typhimurium in mice has been utilized as a model to study typhoid-like disease, due to the characteristics and divergence between both *S.* Typhimurium and *S*. Typhi it is not considered an optimal model. In this sense, different strains of humanized mouse models have been used, which consist of immunodeficient mice that receive human hematopoietic stem cells (Song et al., 2010; Mathur et al., 2012). This model has been developed to study the infection caused by *S.* Typhi and its possible treatment (Song et al., 2010), and it has revealed significant differences in the pathogenesis of typhoid and NTS (Karlinsey et al., 2019).

There are other animal models to study the pathology caused by *S*. Typhimurium, but each of them has some limitations that do not allow a proper extrapolation of the results to humans. On the other hand, studies on human cells or human *in vitro* models are necessary to corroborate the data obtained in animal models, including immortalized cell lines such as HeLa cells as a model of human IECs. In addition, due to the limitations in the isolation of primary intestinal immune cells from human volunteers, most of the studies analyzing immune populations are focused in cell lines and primary cells obtained from human peripheral blood such as THP-1 cells, which are commonly used as a model of human monocytes. Of note, *in vitro* human models lack some intestinal ligands such as commensal microbiota-derived signals, which may play a protective role during *S.* Typhimurium infection, as described in mice (Rivera-Chávez et al., 2016; Jacobson et al., 2018). Thus, these limitations must be considered in order to interpret data from human studies analyzing *Salmonella* infection with different serovars.

### Organoids

As mentioned before, the study of *S.* Typhimurium infection in humans is difficult due to the lack of suitable models. This problem appears especially when it is necessary to study the intestinal colonization occurring in human NTS. In this sense, organoids are a new experimental system developed to study the interaction and colonization of the intestine with enteric pathogens (Wilson et al., 2015). The use of intestinal organoids shows several advantages for the study of *S.* Typhimurium infection, as allows to evaluate the intestinal interaction and invasion process (Co et al., 2019), such as induction of a structural changes of the organoid, the disruption of the tight junctions and also the activation of the NF-κβ inflammatory pathway (Zhang et al., 2014). Also, the organoids display similar organizations (apical, basolateral sides, crypts-like and villus-like regions), epithelial cells interaction, and the differentiation processes observed in the intestine (Zhang et al., 2014; Wilson et al., 2015; Co et al., 2019). Several reports have evaluated the functionality of this system during *S.* Typhimurium infection, showing that the model is suitable. A recent study using both human and mouse enteroids has mapped the full cell cycle of *S.* Typhimurium, revealing that the invasion and colonization of the epithelial barrier and luminal compartment is boosted by TSS-1 and flagellar motility (Geiser et al., 2021). In this case, the use of enteroids has allowed the identification of each consecutive step leading to effective infection of the epithelial barrier and the contribution of particular virulence factors. Furthermore, Holly et al. showed that *S.* Typhimurium infection activated different inflammatory responses depending on whether the organoid was derived from humans or mice (Holly et al., 2020). In mice, caspase-1 is essential to restrict *S.* Typhimurium replication but is dispensable in humans, where it depends on caspase-4 (Holly et al., 2020). Moreover, a recent study has used human organoids to validate transcriptional changes observed in intestinal biopsies infected with *S.* Typhi. These human organoids exhibit altered cytoskeleton reorganization in response to *S.* Typhi, which exploits these changes to favor invasion and immune evasion (Nickerson et al., 2018). Thus, human organoids may be also useful for the study of typhoid serovars. Of note, organoid models have some considerations for their use. The spatial relationship between crypt and villi is not preserved; the concentration of antimicrobial peptides is unknown and the results can vary as the function of microbiota and immune cell interactions are not considered (Nickerson et al., 2018; Holly et al., 2020). As mentioned before, the results depend on their origin.

These models have been crucial in contributing to the understanding of how *S.* Typhimurium infects human IECs, including studies discussed in previous sections. However, we are still finding new genes and functions, implying that we still do not know entirely how *S.* Typhimurium generates the disease, what are the differences between the immune responses and the pathology that this serovar can cause. In this sense, it is crucial to understand the crosstalk between IECs and innate immune cells such as macrophages following *Salmonella* infection in human models (Table 2), which we will review below.

**TABLE 2:** Differences between immune response in human and mice derived cells infected with *S.* Typhimurium.

	Human response	Mouse response	References
*IECs*	Induce the production of IL-1β and IL-23 in human DC and the expression of IL-22 in NCR + ILC3.	Production of IL-8 and different antimicrobial response triggered by IL-22	Pham et al., 2014; Forbester et al., 2018
*Autophagy*	HMGB1 acts as inductor of autophagy in IEC. *S*. Typhimurium has different effector proteins that can block the process, as example, SopF.	HMGB1 acts as inductor of autophagy in IEC. *S*. Typhimurium has different effector proteins that can block the process, as example AvrA	Jiao and Sun, 2019; Xu et al., 2019
*Inflammasome in IECs*	The main pathway is the non-canonical where caspase-1 is required for NLRP3 inflammasome activation and IL-18 secretion	Two pathways: (1) NLRC4 and Caspase-1 with the secretion of IL-1α/β and IL-18. (2) non-canonical NLRP3 and caspase-4 and 5. Activation of NAIP 1–6 proteins, NLRC4	Rioux et al., 2007; Broz, 2014; Knodler et al., 2014a; Zhang et al., 2019
*Inflammasome in macrophage*	Caspase-4 and 5 induce NLRP3 and pyroptosis. Human macrophages express only one type of NAIP protein which recognizes multiple *S*. Typhimurium ligands. In IFN-γ primed macrophages *S*. Typhimurium infection activate caspase-1 and 4 leading pyroptosis. GBP1 sense LPS in IFN-γ primed macrophages and activate pyroptosis dependent of caspase 4	NLRC4 inflammasome activation leading pyroptosis in murine macrophage. GBP are important proteins in the activation of inflammasome. GBP1 sense LPS in IFN-γ primed macrophages	Shenoy et al., 2012; Broz, 2014; Man et al., 2014; Meunier et al., 2014; Pilla et al., 2014; Baker et al., 2015; Kortmann et al., 2015; Schmid-Burgk et al., 2015; Valeria et al., 2017; Fisch et al., 2019a; Santos et al., 2020
*Macrophages*	PGE2 activates caspase-1 and secretion of IL-1β, favoring the macrophage M1 polarization. IL-10 regulates PGE2 production and decrease the antimicrobial ability of these cells. Infected macrophages, secrete exosomes, activating naïve RAW264.7 and promoting the secretion of proinflammatory cytokines.	After infection, SPI-2 effector proteins help in the colocalization between nucleus and hydrolases which promote caspase-11 mediated pyroptosis	Sheppe et al., 2018; Mukhopadhyay et al., 2020; Selkrig et al., 2020
*DC*	It has been observed that infected human DCs preset a suppressive phenotype due to the upregulation of anti-inflammatory molecules as IL-10.	The infection impairs the ability of DC to present antigens through MHC-I/II molecules to T-cells and harming the adaptive immune response. Reduce the capacity of differentiation and poor antigen presentation. Increase number of regulatory T cells.	Tobar et al., 2004, 2006; Van Der Velden et al., 2005; Bueno et al., 2008, 2012; Johanns et al., 2010; Rydström and Wick, 2010; McArthur et al., 2015; Aulicino et al., 2018
*NKT cells*	It is suggested that NKT cell activation is due to the action of TLR10. The activation favors the cross-talk between NKT cells and monocytes and the action of these cells against *S*. Typhimurium.	TCR-independent activation of NKT cell induce the secretion of IFN-γ.	Holzapfel et al., 2014; Bossel Ben-Moshe et al., 2019
*MAIT cells*	*In vitro* activation of human MAIT cells generate the recognition and immune response against infected DCs and B cells. However, the intracellular survival avoids MAIT cells activation.	The activation of MAIT cells generates the secretion of IFN-γ and IL-17, the recruitment other immune cells and the secretion of granzyme A and B against infected cells.	Gold et al., 2010; Kjer-Nielsen et al., 2012

## Immune Responses Against *Salmonella* Infection in Human Models

### Crosstalk Between IECs and Innate Immune Cells During *Salmonella* Infection

One of the first responses against *Salmonella* is mediated by the IECs, which can recognize the cytosolic flagellin and the TTSS-1, inducing an inflammatory response that seeks to inhibit the replication of the bacteria inside the intestinal epithelium and promotes the secretion of cytokines, chemokines and the recruitment of different immune cells. Although an extensive literature has characterized *S.* Typhimurium infection in the mouse gut and the contribution of different immune cells, few studies have addressed these interactions using human cells and typhoid serovars. A recent study has revealed the differential response of human epithelial cells, macrophages, and neutrophils in response to different serovars of *S. enterica*. A human 3-D model consisting of epithelial cells, endothelial cells, fibroblasts, and lymphocytes exhibit differential cytokine patterns in response to *S.* Typhi, Paratyphi A, and Paratyphi B (Salerno-Goncalves et al., 2019). All serovars induce increased expression of IL-8, a chemokine mainly secreted by IECs that recruits neutrophils into infected tissues, whereas *S.* Paratyphi B, and *S.* Typhi induce CCL3 expression, secreted by immune cells. In addition, *S.* Paratyphi A induced higher levels of the pro-inflammatory cytokines IL-6 and TNF-α, whereas *S.* Paratyphi B induced higher levels of IL-1β in a macrophage-dependent manner. In line with this, macrophages are more susceptible to cell death in response to *S.* Paratyphi B, as they probably undergo inflammasome activation, which is accompanied by IL-1β secretion.

Interestingly, neutrophil migration in response to *S*. Paratyphi A and B is higher when macrophages are depleted of the 3-D model, suggesting that macrophages-derived molecules regulate neutrophil migration against these serovars (Salerno-Goncalves et al., 2019). In a similar 3-D model evaluating different strains of *S*. Typhi, including the WT strain Ty2 and the vaccine strain Ty21a, increased levels of IL-1β, IL-8, IL-6, and TNF-α were observed in the culture supernatants in response to both strains. In contrast, Ty21a induced higher levels of the pro-inflammatory cytokine IL-17 and the intestinal mucin Muc2 as compared to Ty2, suggesting that the vaccine strain may induce protective antimicrobial responses (Salerno-Gonçalves et al., 2018). Interestingly, although both strains induce similar cytokines, a PCR array shows that they generate distinctive antimicrobial gene profiles, reinforcing the idea that different strains and serovars of *S. enterica* induce differential immune responses.

Moreover, another 3-D model studying *S.* Typhimurium infection performed transcriptomic analysis of endothelial cells, monocytes, and NK cells, revealing that these cells express IL-8 but also exhibit differential responses to *S.* Typhimurium infection (Schulte et al., 2020). For example, in response to *S.* Typhimurium infection, endothelial cells express high levels of the chemokines IL-6, CXCL6 and CXCL3L1, monocytes exhibit high expression of the pro-inflammatory cytokines IL-1α and IL-1β, and NK cells express the molecules TNFSF4 and IL1-R2, which may regulate vascular immune responses. Although 3-D models are a simplified view of the complex interactions occurring in the gut during infection, they reveal the complex cross-talk between intestinal epithelial and immune cells during infection with different *S. enterica* serovars.

**FIGURE 1:**
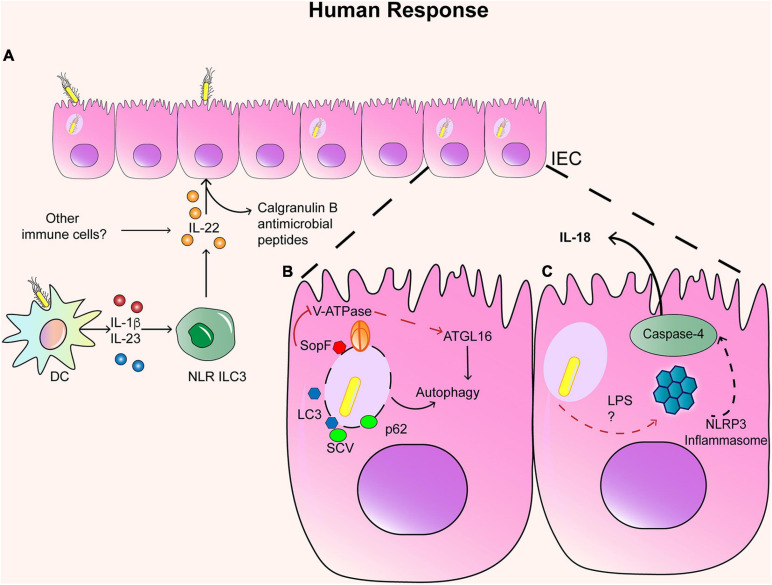
Immune response in IECs. **(A)** Infected IEC respond to IL-22 secreted by NLR ILC3+ cells and other immune cells, which generate the production of Calgranulin B and other antimicrobial peptides. Infected IEC can also activate autophagy and inflammasome. **(B)** In the case of autophagy, SCV can colocalized with LC3 and p62 when the SCV is disrupted. However, SopF can block the interaction between ATGL16 and the SCV, inhibiting autophagia. **(C)** In the case of the activation of the inflammasome, LPS and TTSS-1 can activate NLRP3 inflammasome and the caspase 4 and secretion of IL-18.

As discussed above, IECs are sources of chemokines such as IL-8 and also display antimicrobial responses against *S.* Typhimurium triggered by IL-22 in murine models, secreting different antimicrobial peptides (Figure 1A) (Pham et al., 2014). Indeed, human organoids derived from healthy human pluripotent stem cells infected with *S.* Typhimurium exhibit increased antimicrobial peptide calgranulin B (S100A9), which in turn promoted phagolysosomal fusion in response to IL-22 (Forbester et al., 2018). These protective responses are lost in organoids from patients with a genetic mutation in the IL-22 receptor (Forbester et al., 2018). Thus, IL-22 also regulates anti-*S.* Typhimurium responses in human IECs. The primary innate producers of IL-22 in murine models are type 3 innate lymphoid cells (ILC3), and several studies have addressed their relevance during early immune response against *S.* Typhimurium infection in mice. A recent study has confirmed that *S*. Typhimurium stimulates IL-1β and IL-23 production in human DC and subsequently induces IL-22 expression in human colonic NCR + ILC3 (Figure 1A) (Castleman et al., 2019). Therefore, the IL-22-induced antimicrobial pathway seems to be conserved in human intestinal cells, inducing protective responses against *S.* Typhimurium. However, it remains unclear whether this pathway is conserved in humans during infection with either *S.* Typhi, *S.* Paratyphi, or *S*. Enteritidis.

Moreover, *in vitro* studies have shown that both human epithelial cells and immune cells induce programmed cell death and antimicrobial responses during *S.* Typhimurium infection. Thus, we will explore recent findings on these topics.

### Programmed Cell Death as a Defense Mechanism Against *Salmonella* Infection

#### Autophagy in Epithelial Cells

Autophagy has been described as an important host response to eliminate *S.* Typhimurium from IEC in murine models (Figure 1B) (Benjamin et al., 2013). Once *S.* Typhimurium enters host cells, it is targeted by different host proteins including ubiquitins, LC3 and p62, which leads to autophagosome formation and subsequent cell death (Wang et al., 2018). Indeed, *S.* Typhimurium co-localizes with LC3 in HeLa cells, in a mechanism dependent on T3SS-1, suggesting that bacterial-induced vacuolar damage can trigger autophagy (Birmingham et al., 2006). This is consistent with a recent study, which describes that a V-ATPase, responsible for acidification in vacuoles, acts as a sensor of vacuolar damage caused by *S.* Typhimurium (Xu et al., 2019). Furthermore, the V-ATPase recruits the autophagy-related 16-like 1 (ATG16L1) protein, which is crucial for bacterial autophagy (also known as xenophagy). A knockdown of ATG16L1 in HeLa cells demonstrated that this protein is required for autophagy against *S.* Typhimurium (Rioux et al., 2007). In addition, genetic variants of ATG16L1 are linked to Crohn’s disease, and human IEC lines HeLa and Caco-2 expressing those variants exhibit impaired autophagy against *S.* Typhimurium (Kuballa et al., 2008). This is consistent with the evidence in murine models, which suggest that *S.* Typhimurium infection can increase susceptibility to IBD (Schultz et al., 2018).

Recent studies in the human cell line HTC116 have also revealed host and bacterial molecules involved in regulating autophagy in response to *S.* Typhimurium. Knockdown studies in this cell line have shown that the intracellular protein high mobility group box 1 (HMGB1) acts as an inductor of autophagy in epithelial cells by preventing STAT3 activation in infection assays with *S.* Typhimurium. Further experiments in murine models proved a protective role of HMGB1 in *Salmonella* infection (Zhang et al., 2019). Conversely, different *Salmonella* serotypes express effector molecules that can block autophagy. *S.* Enteritidis can suppress autophagy in HTC116 cells by reducing the expression of the regulator Beclin-1 in a mechanism dependent on the T3SS-1-effector molecule AvrA and induces less weak autophagic activity (Jiao and Sun, 2019). This mechanism was further demonstrated *in vivo* in murine infection. Although *S.* Typhimurium seems to induce robust autophagy, it can block it in a T3SS-1-dependent manner. Assays in HeLa cells revealed that the T3SS-1 effector SopF blocks *S*. Typhimurium-induced autophagy. In particular, SopF can ADP-ribosylate the V-ATPase, preventing its interaction with ATG16L1, blocking bacterial autophagy but not canonical autophagy (Figure 1B) (Xu et al., 2019). Therefore, manipulation of bacterial autophagy is an attractive mechanism for potential drug development against *Salmonella* infection.

#### Inflammasome Activation in IECs

Several reports in murine models have shown that IECs can also activate the inflammasome, which is a multi-protein complex critical for the clearance of *S.* Typhimurium and host defense (Bierschenk et al., 2017). Pathogen recognition promotes two ways of inflammasome activation: (1) the NLRC4 inflammasome and caspase-1 (Sellin et al., 2014; Rauch et al., 2017) (2) and the non-canonical pathway activation of NLRP3 inflammasome and caspase-4 and caspase-5 (the human orthologs of caspase-11) (Knodler et al., 2014a). Evidence in murine models indicates that the early recognition of *S*. Typhimurium infection (<36 h post-infection) (Broz, 2014) generates the activation of NAIP 1–6 proteins, NLRC4 inflammasome, and caspase-1, which restrict this replicative niche due to the expulsion of the infected IECs from the mucosa (Sellin et al., 2014). It has been shown that other inflammasomes, such as NLRP3 or NLRP6, are dispensable during *S.* Typhimurium infection in murine models, as well as the downstream cytokines of NLRC4 as IL-1α/β and IL-18 (Sellin et al., 2014). However, caspase-4 is required for the induction of IL-18 in response to *S.* Typhimurium infection, whereas caspase 1 contributes to the release of IL-1β (Knodler et al., 2014a), indicating that the non-canonical inflammasome also contributes to immunity against *Salmonella* (Figure 1C).

Indeed, the non-canonical pathway seems to be more relevant in human immunity against *S.* Typhimurium. Infection assays in polarized human colonic epithelial cells (C2Bbe1) have shown that the caspase-4 but not caspase-1 is required for inflammasome activation and subsequent secretion of IL-18 in an LPS-dependent manner (Knodler et al., 2014a). As mentioned before, the requirement of caspase-4 has been recently confirmed using human enteroid monolayers, whereas mouse organoids exhibit opposite results (Holly et al., 2020), which is consistent with previous data from murine models (Sellin et al., 2014). Thus, human IECs undergo caspase-4-dependent inflammasome activation during *S.* Typhimurium infection, although evidence of the signaling pathways regulating their activation in human IECs is still required.

#### Inflammasome Activation in Human Macrophages

**FIGURE 2:**
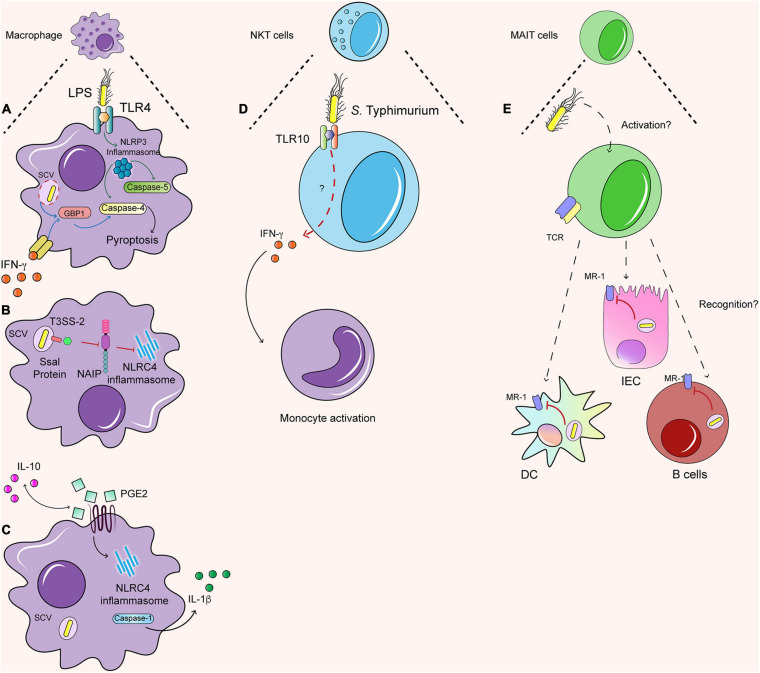
Immune response against *S.* Typhimurium in human cells. **(A)** LPS dependent NLRP3 inflammasome induce the caspase 4/5 dependent pyroptosis. Further, the IFN-γ primed macrophages generate the same dependent pyroptosis process, but with the activation of GBP1. **(B)** In macrophages Ssal, an effector protein secreted by TTSS-2, can inhibit the activation of NLRC4 by the NAIP proteins. **(C)** IL-10 regulate the production of PGE2 and the production of IL-1β dependent of NLRC4 inflammasome. **(D)** NKT cells are able to recognize *S.* Typhimurium possible by TLR 10, which by a non-identify pathway lead the secretion of IFN-γ that activate monocytes. **(E)** It is still unknown how MAIT cells are activated. When are activated, they can recognize infected DC, IEC, and B cells and secrete granzyme. However, the *S*. Typhimurium inside SCV can avoid the load into MR-1 and in this sense avoid the recognition by MAIT cells as well.

Macrophages and other immune cells also undergo inflammasome activation (Figure 2), which is accompanied by the induction of a programmed cell death pathway known as pyroptosis. Evidence in murine macrophages and the human monocytic cell line THP-1 indicates that both inflammasomes are redundant against *S.* Typhimurium infection, both leading to pyroptosis (Broz et al., 2010; Man et al., 2014). Similar to IECs, both caspase-4 and caspase-5 also induce the NLRP3 inflammasome in macrophages by detecting LPS and *S.* Typhimurium in a TLR-4-dependent manner, as shown in caspase 4- and caspase 5- deficient THP-1 cells (Figure 2A) (Baker et al., 2015; Schmid-Burgk et al., 2015). In addition, a similar model of caspase-4 silencing in human monocyte-derived macrophages (HMDM) showed that caspase-4 was required for inflammasome activation in a T3SS-1-dependent manner (Casson et al., 2015). In contrast to the mouse canonical NLRC4 inflammasome, human macrophages express a single NAIP protein, which recognizes multiple *S.* Typhimurium ligands including flagellin and the T3SS-1 inner protein PrgJ but is evaded by the T3SS-2 protein SsaI (Figure 2B) (Kortmann et al., 2015; Valeria et al., 2017). This is in line with recent evidence indicating that *S.* Typhimurium SPI-2 is required to downregulate inflammasome responses in both THP-1 and HMDM, as cells are infected with a SPI-2-deficient strain exhibit enhanced NLPR3 and NLRC4 inflammasome responses (Bierschenk et al., 2019). Thus, T3SS-1 effector proteins induce the inflammasome, whereas T3SS-2 effector proteins can suppress it, allowing *S.* Typhimurium survival.

IFN-γ is also an important signal regulating caspase-dependent response against *S.* Typhimurium in human macrophages. Humans challenged with *S.* Typhi exhibit increased production of types I and II interferons (Blohmke et al., 2016), including IFN-γ, which is also produced by different innate and adaptive sources in murine models during *Salmonella* infection (Thiemann et al., 2017). IFN-γ-primed macrophages undergo both caspase-1 and caspase-4-dependent pyroptosis in THP-1 cells and HMDM in response to *S.* Typhimurium (Fisch et al., 2019a). Different studies in mice have shown that interferon-inducible protein guanylate binding proteins (GBP) are important in activating inflammasome responses against *S.* Typhimurium, as mice deficient in the GBP cluster or GBP5 exhibit decreased caspase 11-dependent pyroptosis (Figure 2A) (Shenoy et al., 2012; Meunier et al., 2014; Pilla et al., 2014). In line with this evidence, recent work has shown that GBP1 acts as an LPS sensor, targeting *S.* Typhimurium just after SCV rupture in IFN-γ-primed human macrophages and mediating caspase-4 dependent pyroptosis (Fisch et al., 2019a; Santos et al., 2020). This mechanism has also been described in human organoids primed with IFN-γ, in which GBP1 can recruit GBP2, GBP3, and GBP4 in an LPS-dependent manner, controlling caspase-4 recruitment and activation of the non-canonical inflammasome (Kutsch et al., 2020; Santos et al., 2020; Wandel et al., 2020). In addition, caspase-1 regulates negatively GBP1 function in human macrophages, preventing caspase-4-dependent pyroptosis and regulating cell death in *S.* Typhimurium infection (Fisch et al., 2019b). Therefore, interferon-induced responses trigger *S.* Typhimurium sensing and initiation of caspase-dependent responses in human macrophages. In addition to programmed cell death mechanisms, human macrophages and other innate immune cells display important anti-microbial functions during *Salmonella* infection, which will be discussed in the section below.

### Novel Anti-*Salmonella* Functions in Human Innate Cells

#### Proteomics in Infected Human Macrophages

Macrophages are the primary carriers of *S.* Typhimurium infection, and thus recent studies have explored how the bacteria impact the proteome of macrophages. A proteomic study in the murine macrophage cell line RAW264.7 has revealed new regulators of the non-canonical inflammasome. The lysosomal hydrolases cathepsins are enriched in the nuclei following *S.* Typhimurium infection in a SPI-2-dependent manner and subsequently promote caspase-11-mediated-pyroptosis in mice (Selkrig et al., 2020). In human macrophages, proteomic studies have revealed a new regulator of the canonical inflammasome. Increased levels of eicosanoids were detected using quantitative mass spectrometry in *S.* Typhimurium-infected THP-1 cells. In particular, prostaglandin 2 (PGE2) production activates caspase-1 and IL-1β production in infected macrophages and promotes their polarization toward a M1 phenotype (Sheppe et al., 2018). Of note, a recent study has shown that IL-10 regulates the production of PGE-2 in human macrophages differentiated from pluripotent stem cells lacking the IL-10 receptor. These macrophages exhibit defective *S.* Typhimurium killing, which was attributed to the increased production of PGE2 in response to *S.* Typhimurium in the absence of IL-10 signaling (Figure 2C) (Mukhopadhyay et al., 2020). Therefore, PGE2 plays an important yet complex role in regulating the inflammasome and anti-bacterial responses in macrophages.

Moreover, other proteomic studies have identified novel mechanisms in the macrophage response to *S.* Typhimurium infection. A genome-wide CRISPR approach in THP-1 cells identified 183 genes related to host defense, including genes related to actin dynamics, metabolic pathways, and calcium transport in response to *S.* Typhimurium infection (Yeung et al., 2019). In particular, this study identified NHL-repeat-containing protein 2a (NHLRC2) as a regulator of *Salmonella* phagocytosis and the actin cytoskeleton. Mutant THP-1 macrophages lacking NHLRC2 exhibit a hyper-inflammatory profile but display an abnormal morphology and are unable to phagocytose *S.* Typhimurium. Similarly, another study analyzed the extracellular proteome of THP-1 cells using mass spectrometry and described the secretion of exosomes from *S.* Typhimurium-infected macrophages (Hui et al., 2018). These exosomes from infected macrophages trigger the activation of naïve RAW264.7 macrophages, promoting the secretion of pro-inflammatory cytokines such as TNF-α, G-CSF, GM-CSF, and RANTES. Therefore, proteomic analyses of human macrophages have revealed novel antimicrobial molecules (Hui et al., 2018).

#### Single-Cell Sequencing in Innate Cells During *S.* Typhimurium Infection

Although multiple innate cells have been associated with protective effects against *Salmonella* infection in murine models, evidence exploring their relevance in humans is scarce. Recent transcriptomic analysis using single-cell sequencing technologies has described the dynamics and heterogeneous responses of innate cells during *S.* Typhimurium infection. Extensive literature has characterized how *Salmonella* infects and exploits intestinal DC function in murine models in order to disseminate systemically, but few studies have explored how *Salmonella* modulates human DCs. Aulicino et al. (2018) analyzed the differential responses of monocyte-derived DC in response to either the non-invasive strain LT2 and the invasive serotype ST313. Compared to LT2, ST313 exploits DC responses to avoid immune detection by reprogramming the transcriptome of infected and bystander DC. ST313-infected cells upregulate the anti-inflammatory molecules IL-10 and downregulate the activation markers CD83, CD86, and antigen presentation, suggesting that they develop a more suppressive phenotype. In addition, bystander DCs show hyper-inflammatory responses, which may recruit adaptive immune cells against uninfected cells and facilitate *S.* Typhimurium survival (Aulicino et al., 2018). Therefore, this study reveals the heterogeneity of human DC function in response to the invasive ST313 that could facilitate its dissemination and survival in human disease (Aulicino et al., 2018). Although there is evidence that *S.* Typhi can also manipulate human DC function (Bueno et al., 2008), more extensive transcriptomic analysis need to be performed in order to reveal serovar-specific mechanisms.

Furthermore, single-cell sequencing of PBMCs from humans challenged *ex vivo* with *S.* Typhimurium has revealed the dynamics of immune populations during infection. Monocytes exhibit different distinctive changes after infection, indicating bacteria internalization and changes in monocyte subsets, suggesting that some of them might undergo cell death whereas others resemble M1 macrophages. In addition, a particular cluster of NKT cells emerges from CD8^+^ T cells and exhibits distinctive changes in their gene signature following infection. In addition, individuals with a polymorphism in the TLR10 gene exhibit decreased activation of NKT cells and reduced IFN-γ mediated cell to cell signaling between NKT cells and monocytes against *Salmonella*, indicating that NKT-monocyte cross-talk is important during *S.* Typhimurium infection (Figure 2D) (Bossel Ben-Moshe et al., 2019).

NKT cells recognize unconventional non-peptide ligands (in this case, glycolipids) derived from some Gram-negative bacteria bound to CD1d molecules that, unlike MHC, are highly invariant (Dellabona et al., 1994; Cox et al., 2009). However, the activation can be mediated by three different mechanisms: direct TCR activation; independent TCR activation (which is mediated by APC and leads to cytokine secreted, as IL-12 or IL-18); or independent and dependent TCR activation (a combination of the previously mentioned mechanism) (Reilly et al., 2010; Holzapfel et al., 2014). Murine models of *S.* Typhimurium infection exhibit TCR-independent NKT activation, suggesting that cytokine signaling is sufficient to activate NKT cells to produce IFN-γ (Holzapfel et al., 2014). The evidence shown by Bossel Ben-Moshe et al. (2019) suggests that TLR-10 may be involved in human NKT activation during *Salmonella* infection, but further research is required to confirm this idea.

#### MAIT Cells Protective Responses Against *Salmonella*

In addition to NKT cells, *S.* Typhimurium encounters other innate-like T cells with innate and adaptive properties: the mucosal associated invariant T (MAIT) cells (Figure 2E). These cells are characterized by the expression of a semi-invariant TCR (Vα19-Jα33) (Tilloy et al., 1999) and are highly conserved between mouse and human. MAIT cells respond against metabolites products of the riboflavin pathway of Gram-negative and positive bacteria, which are presented by antigen-presenting cells in non-classical class I antigen-presenting molecules: MHC-like protein 1 (MR-1) (Treiner et al., 2003; Kjer-Nielsen et al., 2012). The immune response of MAIT cells generates the secretion mainly of IFN- and IL-17, both acting as antibacterial molecules that also recruit other immune cells and have cytotoxic effects against infected cells, secreting granzyme A and B (Le Bourhis et al., 2013).

*Salmonella* Typhimurium can activate human MAIT cells *in vitro* (Le Bourhis et al., 2013) and generate an immune response to infected-DCs and B cells (Gold et al., 2010; Kjer-Nielsen et al., 2012; Salerno-Goncalves et al., 2014). When MAIT cells are activated, they develop a cytotoxic function against infected cells. However, this recognition is impaired because the bacteria remain within SCV, preventing the antigen load into MR-1 (Le Bourhis et al., 2013). This effect is observed with *S*. Typhimurium WT and mutants of the SPI-1 or SPI-2, suggesting that intracellular *S*. Typhimurium prevents MAIT cell activation against infected epithelial cells (Le Bourhis et al., 2013). However, increasing evidence suggests that MAIT cell adaptive immune response may be important in the control of *Salmonella* infection. Although human volunteers challenged with *S.* Paratyphi A exhibit lower frequencies of circulating MAIT cells (Howson et al., 2018), an increased proportion of MAIT cells are activated, exhibiting clonal expansion and high avidity to the riboflavin metabolite ligands. This evidence suggests that MAIT cells may be related to the adaptive immune response against *Salmonella*, as the expanded clonotypes could be protective against re-infection. In fact, human volunteers challenged with a low dose of WT *S.* Typhi also exhibited increased frequency of activated CD8^+^ MAIT cells, which also exhibited increased expression of proliferation and intestinal-homing markers. Interestingly, volunteers with increased susceptibility to the infection displayed reduced frequency of circulating MAIT cells, associated with increased exhaustion and apoptosis (Salerno-Goncalves et al., 2017). In line with these findings, a murine pulmonary model of *S.* Typhimurium infection induces the activation of MAIT cells in an IL-23-dependent manner, enhancing ICOS expression and induces a TH17 phenotype in MAIT cells. In addition, in the same study, vaccination using IL-23 and the riboflavin ligand 6- d -ribitylaminouracil (5-OP-RU) activates MAIT cells and protect against *Legionella* infection in mice, suggesting that MAIT cells may provide protection against other pathogens such as *Salmonella* (Wang et al., 2019). Moreover, recent evidence indicates that the ST313 strains D23580 and D3771 are able to evade MAIT cell recognition in both healthy and immunocompromised individuals in a mechanism dependent on the overexpression of *ribB.* This enzyme is involved in the riboflavin pathway and leads to low intracellular levels of flavin mononucleotide, a ligand for MAIT cell activation. In contrast, other ST313 strains such as U2, U5, and D25248, the non-invasive strain *S.* Typhimurium 4/74, *S.* Typhi and *S.* Paratyphi induced potent MAIT cell activation (Preciado-Llanes et al., 2020). Together, this evidence supports an important role of MAIT cells in protection against different serovars of *Salmonella*, with the potential of generating adaptive responses protective against re-infection.

## Conclusion

*Salmonella enterica* generates two types of diseases: non-typhoidal and typhoidal disease. However, in the case of NTS disease caused by *S.* Typhimurium, depending on the immune response of the patient, NTS can generate complications during the disease such as carriage and iNTS. In this sense, iNTS generate more deaths than typhoidal serovars, the invasive serotype ST313, which as in the case of *S*. Typhi, has been suffered a host adaptation to the human and especially to immunocompromised ones causing, for instance, less intestinal invasion and inflammation.

In line with this, most of the studies performed have addressed the response exhibited in mice during *S.* Typhimurium infection, but studies in human hosts have been limited to human cell lines to corroborate the discoveries made in animal models. Several *Salmonella* virulence genes perform similar functions in both models (human and mouse cells). However, some effector proteins such as Ssel or SseK seem to have different functions in human and mouse cells, but this difference is just related to the type of human cell lines utilized in the studies and requires in-depth investigation.

Similarly, the immune response generated by different serovars of *S. enterica* exhibit similar mechanisms in mouse and human models in order to control the infection. However, the contribution of the canonical and non-canonical pathways of inflammasome activation differ in mice and humans, thus there is still much work to do to understand the regulation of this pathway in human cell lines. Moreover, transcriptomic and proteomic analyses have revealed novel antimicrobial pathways in human macrophages, regulating the infection in these cells and also shown the complex immune responses displayed by other innate cells such as DC, NKT, and MAIT cells, with essential roles during the immune response against *S.* Typhimurium infection. However, the knowledge about mechanisms mediated by these immune cells in humans is still scarce and is necessary to perform more studies to understand how they respond against the infection, and what are the differences between human and mice that lead to different pathologies.

Finally, it is imperative to develop a proper model to study all the differences between several serovars of *S. enterica* and between mouse and human immune response against *S.* Typhimurium infection. Also, it is required a model that integrates the interaction with cells of the immune system, together with the human microbiota, resembling the interactions present *in vivo* with other types of intestinal cells or with soluble factors that can determine the pathology and the immune response. Currently new techniques have been tested and used, allowing the global characterization of the transcriptome and proteome during infection, which has improved our knowledge; however, we continue discovering new functions or interactions for previously known virulence proteins or finding new activation pathways during the infection, which implies that we still do not know entirely how typhoid and non-typhoid serovars generates the disease and all the factors involved, especially in human infection.

## Author Contributions

All authors listed have made a substantial, direct and intellectual contribution to the work, and approved it for publication.

## Conflict of Interest

The authors declare that the research was conducted in the absence of any commercial or financial relationships that could be construed as a potential conflict of interest.
